# Higher expression of* SOX1*, miR-155, and miR-21 in the colostrum of SARS-CoV-2-infected mothers

**DOI:** 10.1038/s41598-026-49964-4

**Published:** 2026-04-20

**Authors:** Paulina Gil-Kulik, Dominika Przywara, Michał Mitrus, Alicja Petniak, Bartosz Kondracki, Urszula Szymanowska, Rafał Szymanowski, Monika Czuba, Adrianna Kondracka, Janusz Kocki

**Affiliations:** 1 Department of Clinical Genetics, Medical University of Lublin, 11 Radziwillowska Str., 20-080 Lublin, Poland; 2 Doctoral School, Medical University of Lublin, 20-093 Lublin, Poland; 3 Student Scientific Society of Clinical Genetics, Medical University of Lublin, 11 Radziwillowska Str., 20-080 Lublin, Poland; 4 Department of Obstetrics and Pathology of Pregnancy, Medical University of Lublin, 11 Staszica Str., 20-081 Lublin, Poland; 5 Department of Cardiology, Medical University of Lublin, 20-059 Lublin, Poland; 6 Department of Biochemistry and Food Chemistry, University of Life Sciences, 8 Skromna Str., 20-704 Lublin, Poland

**Keywords:** microRNA, miRNA, COVID-19, Breast milk, Colostrum, *SOX1*, miR-21, miR-155, Biochemistry, Biomarkers, Diseases, Immunology, Medical research, Molecular biology

## Abstract

The aim of this study was to assess the impact of SARS-CoV-2 infection during pregnancy on the molecular profile of human milk and to identify associations between the expression of regulatory genes and clinical parameters of newborns. The study included a group of 19 women who had COVID-19 during pregnancy and a healthy control group of 21 women. Expression levels of miR-21, miR-155, and the transcription factor *SOX1* were determined in human milk using qPCR. The correlation of *SOX1* expression with parameters in cord blood and infant blood was also analyzed. It was shown that maternal SARS-CoV-2 infection (during pregnancy) is associated with significantly higher levels of miR-21, miR-155, and *SOX1* expression in human milk. Furthermore, strong statistical correlations were found in the study group: *SOX1* expression was positively correlated with C-reactive protein (CRP) levels and lactate concentration (LAC) in arterial umbilical cord blood, while negatively correlated with glucose levels. Our research shows that SARS-CoV-2 infection during pregnancy induces changes in the molecular composition of early breast milk, promoting immunomodulatory (miR-155) and regenerative (miR-21, *SOX1*) molecules. Post-COVID-19 breast milk serves as an active biochemical communication system, providing the newborn with signals that may modulate adaptive and immune responses to the unfavorable intrauterine environment.

**Supplementary Information:**

The online version contains supplementary material available at 10.1038/s41598-026-49964-4.

## Introduction

Coronavirus disease 2019 (COVID-19), caused by SARS-CoV-2, is associated with a wide range of long-term complications, including neurological changes such as white matter damage and axonal injury^1–5^. These effects are particularly concerning in pregnancy, as maternal viral infections can impair fetal neurodevelopment^6–10^. SARS-CoV-2 may also affect placental integrity, increasing the risk of preeclampsia, preterm birth, and low birth weight^9–14^.

Human milk provides all essential nutrients for exclusive infant feeding during the first six months of life and remains a key component of a child’s diet in subsequent stages^15,16^. Beyond nutrition, it serves as a dynamic biochemical communication system, delivering protective proteins, stem cells, and immunomodulatory microRNAs (miRNAs)^17^. The composition of human milk is sensitive to maternal health^18^. SARS-CoV-2 infection during pregnancy has been shown to alter the profiles of proteins and lipids involved in the inflammatory response^19–21^. Importantly, breast milk from mothers who have had COVID-19 may contain lower levels of lactoferrin and secretory immunoglobulin A^20^. This reduction likely reflects a reconfiguration of the maternal immune system following the systemic challenge of infection, potentially impacting passive immunity transferred to the infant^20^. Breast milk has an important function in the development of the newborn. Cells from the milk penetrate the child’s intestinal wall and reach various organs, including the brain. The fact that the RNA of the SARS-CoV-2 virus can be detected in breast milk in the first few days after birth may be relevant to the properties of milk^22,23^.

In this context, miR-155 and miR-21 have emerged as key immunoregulatory molecules in human milk. These miRNAs help shape the infant’s immune response and inflammatory tone, which is particularly important in the context of inflammation and antiviral defense associated with COVID-19^24–28^. Both miR-155 and miR-21 are key players in the SARS-CoV-2-induced cytokine storm and are known to modulate the expression of SOX family genes^29^. SOX1, a member of the SOX B1 group, is a key regulator of embryonic development and neurogenesis^30–32^. Although human milk is known to contain stem cells expressing pluripotency factors^33–36^, SOX1 expression in this biological fluid may represent a compensatory maternal signal to support neonatal neuronal maturation and cytoprotection in response to the stress of infection^37–39^.Furthermore, while the associations between SOX1 antibodies and neuroimmune or paraneoplastic syndromes are recognized^40,41^, the interplay between viral infection and *SOX1* expression in colostrum has not yet been confirmed.

Despite its potential importance, the interplay between maternal SARS-CoV-2 infection and the SOX1-miRNA axis in colostrum remains to be fully elucidated. Therefore, the aim of our study was to assess the expression of SOX1, miR-155, and miR-21 in human milk cells collected from women who had COVID-19 during pregnancy. By analyzing this axis, we aimed to elucidate whether maternal viral infection induces a coordinated miRNA-SOX1 response in human milk that could potentially influence the transcriptomic structure and development of the infant^42^.

## Material and methods

### Ethics approval

The study was conducted on the basis of a protocol approved by the Bioethics Committee of the Medical University of Lublin (no. KE-0254/88/04/2022). Each patient gave written consent to collecting material, conducting tests and publishing the test results.

### Patient qualification

Patients were qualified for the study by a doctor from the Department of Obstetrics and Pathology of Pregnancy, USK No. 1 in Lublin, Poland, on the basis of a medical interview and the results of available laboratory tests.

The inclusion criteria for the study group were: a non-smoking patient who did not use stimulants, pregnancy completed on time, by vaginal birth (VB) or cesarean section (CC) delivery without complications, the newborn was in good condition, with proper adaptation, the patient had a SARS-CoV-2 infection during pregnancy, confirmed by a positive antigen test result or PCR (infection in the first, second or third trimester of pregnancy).

The following patients were qualified for the control group: a non-smoker, not using stimulants, pregnancy completed on time, by ND or CC delivery without complications, the newborn in good condition, with proper adaptation, the patient denies symptomatic SARS-CoV-2 infection and other respiratory infections during pregnancy.

Information was collected from patients from both groups regarding the possible occurrence of concomitant diseases during pregnancy, such as diabetes, hypothyroidism, medications used during pregnancy, results of basic laboratory tests, and vaccinations against COVID-19 during pregnancy. Before collecting the material, the patient completed a detailed questionnaire regarding the course of the current pregnancy and delivery, the number and course of previous pregnancies and deliveries, age, height, body weight, BMI before pregnancy, current BMI, medications and supplements used during pregnancy, infections occurring during pregnancy, using stimulants.

#### Downloading the material

On the third day after delivery, after qualifying for the study by the attending physician and completing the questionnaire, approximately 5 ml of milk was collected from patients in a sterile container. Milk was collected in the morning, approximately two hours after the meal. Immediately after collection, the sample was transported to the Department of Clinical Genetics of the Medical University of Lublin, where the isolation of milk cell fractions began.

### Colostrum cell isolation

The material for the study was the cellular fraction of breast milk isolated from 5 ml of whole milk by centrifugation (centrifugation conditions: 20 min, 805 g). After isolation, the cells were washed twice with PBS-buffered saline (Biomed, Poland). In this way, a heterogeneous cellular fraction of breast milk was obtained, proposed by Hassiotou^43^, containing breast milk stem cells, and then RNA was isolated from these cells.

#### RNA isolation

Total cellular RNA was isolated from the obtained cell fraction using a column technique using the mirVana™ miRNA isolation kit (Invitrogen by ThermoFisherScientific, Vilnius, Lithuania), according to the manufacturer’s recommendations. An on-column DNase treatment was included to ensure total removal of genomic DNA. The quality, quantity and purity of the isolated RNA extract were assessed spectrophotometrically (Nanodrop 2000c, Thermo Fisher Scientific, Waltham, MA, USA). RNA integrity was further evaluated using the Agilent TapeStation System. The mean RNA Integrity Number (RIN) was 7.49 ± 0.67 (range: 6.6–8.6) for the COVID-19 group and 7.30 ± 0.64 (range: 6.7–8.5) for the control group. These values confirmed that the RNA quality was adequate for subsequent analyses, ensuring that the observed expression differences were not driven by RNA degradation.

#### Reverse transcription

The isolated RNA was used for a reverse transcription reaction performed using commercially available ThermofisherScientific kits: TaqMan MicroRNA Reverse Transcription Kit with primers appropriate for probes used to assess microRNA expression and the High-Capacity RNA-to-cDNA kit. The reverse transcription reaction was performed according to the reagent manufacturer’s instructions.

The reverse transcription reaction for miRNA was performed with the TaqMan MicroRNA Reverse Transcription Kit and miRNA-specific stem-loop primers (Applied Biosystem, Vilnius, Lithuania), adding 10 ng total RNA dissolved in 5 μl nuclease-free water to 10 μl RT Reaction Mix: 3 μl (5 × RT Primer); 1.5 μl (10 × Reverse Transcription Buffer); 1 μl (MultiScribe Reverse Transcriptase, 50 U/µL); 0.15 μl (100 mM dNTPs (with dTTP)); 0.19 μl (RNase Inhibitor, 20 U/μL); 4.16 μl (Nuclease-free Water), and then the reverse transcription reaction was performed in a Vieriti Thermal Cycler (Applied Biosystems, Foster City, CA, USA), according to the scheme: phase I 16 °C–30 min; phase II 42 °C–30 min and stop reaction 85 °C, 5 min.

For the *SOX1* and *GAPDH* control gene, 1 µg of total RNA was used to transcribe cDNA using the High-Capacity cDNA Reverse Transcription Kit with RNase Inhibitor (Applied Biosystem, Vilnius, Lithuania). A cDNA synthesis was performed on a Veriti Thermal Cycler (Applied Biosystems, Foster City, CA, USA) in the following order: phase I 25 °C, 10 min; phase II 37 °C, 120 min; phase III 85 °C, 5 min; phase IV 4 °C. The resulting cDNA was used for real-time PCR.

#### qPCR expression assessment

The expression of microRNA and *SOX1* was assessed by qPCR in the StepOne Plus system (Applied Biosystems, Waltham, MA, USA). Commercially available TagMan probes (Applied Biosystems, Pleasanton, USA; ThermofisherScientific) were used for determinations: has-miR-21-3p (ID 002438), Mature miRNA Sequence: CAACACCAGUCGAUGGGCUGU; hsa-miR-155-5p (ID 002623) Mature miRNA Sequence: UUAAUGCUAAUCGUGAUAGGGGU; for *SOX1*: Hs01057642_s1 (NM_005986.2); As an endogenous control, the miRNA: RNU48 (ID 001006) (GATGACCCCAGGTAACTCTGAGTGTGTCGCTGATGCCATCACCGCAGCGCTCTGACC) was used, and the gene: GAPDH: Hs99999905_m1 (NM_002046.7).

qPCR reactions were performed, including 4.5 µl (0.67 µl of cDNA from the reverse transcription reaction together with 3.83 µl of ultrapure water, free from RNase and DNase) and 0.5 µl of a probe specific for the tested gene or miRNA and 5 µl of TaqMan Gene Expression Master Mix (Applied Biosystems, Vilnius, Lithuania). PCR reaction scheme: initial denaturation for 10 min at 95 °C, then 40 cycles: 15 s at 95 °C and 60 s at 60 °C. Relative expression levels were assessed using the formula RQ = 2^-ddCt^. To normalize the expression of the studied microRNAs and the *SOX1* gene, the CT value was determined for each sample relative to the reference gene (*RNU48*/*GAPDH*). Final gene expression was determined relative to the sample used to calibrate the entire experiment. The relative expression level (RQ) was calculated according to the method of Livak^44^ using Expression Suite Software version 1.3 (LifeTechnologies).

#### Normalization and validation

In accordance with the Minimum Information for Publication of Quantitative Real-Time PCR Experiments (MIQE) guidelines^50^, each sample was assessed three times, a negative control was used, the qPCR evaluation technique had been previously validated, the reference genes used for the experiment were selected as the most stable among the three genes determined in the experiment. To ensure accurate normalization, the expression stability of three candidate endogenous controls (*ACTB, B2M,* and G*APDH*) was initially evaluated^33,36,43,45^. *GAPDH* was identified as the most stable reference gene across all experimental groups (*p* > 0.05) and was thus utilized for mRNA normalization. For microRNA normalization, RNU48 was selected based on its high stability in human milk cell fractions, as previously validated in our preliminary microarray screenings. Consequently, *GAPDH* and RNU48 was utilized as the sole endogenous control for the final calculation of relative expression levels (RQ). All procedures were performed in accordance with the recommendations of the manufacturer of the reagents and equipment used.

#### Statistical analysis

Statistical analysis was performed using the Statistica program. The normality of data distribution was assessed using the Shapiro–Wilk test. Since all log-transformed RQ values (logRQ) followed a normal distribution (*p* > 0.05 in all cases), parametric methods were applied. Specifically, the Student’s t-test and Pearson correlations were used to assess differences between the study groups. The significance level was *p* < 0.05.

Due to the exploratory nature of this pilot study and the relatively small sample size, correlations were reported without correction for multiple comparisons. Consequently, these findings should be interpreted as hypothesis-generating rather than confirmatory.

## Results

### Study population

**Fig. 1:**
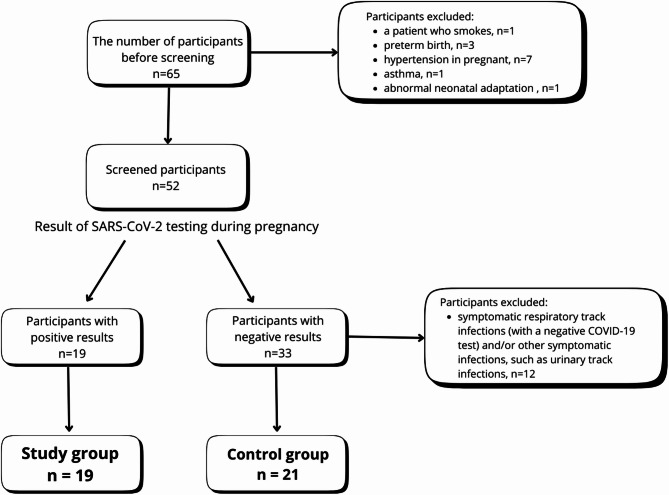
Flow chart of the study illustrating participant selection and assignment.

A total of 40 patients hospitalized after childbirth at the Department of Obstetrics and Pathology of Pregnancy, USK No. 1 in Lublin, Poland were qualified for the study, of which 21 were included in the control group, while 19 patients who had COVID-19 confirmed by a test during pregnancy were qualified for the study group. Flow chart of the study is presented in Fig. 1. The age of the patients ranged from 18 to 43 years. Detailed characteristics of the studied patients are presented in Tables 1 and 2.

**Table 1:** Characteristics of the study group. Parameters characterizing the mother, child and selected parameters of umbilical cord blood.

Parameter	Group of patients												*p*-value
	Control group						Patients with COVID-19 during pregnancy						
	N*	Mean	Median	Min	Max	SD	N*	Mean	Median	Min	Max	SD	
Mother parameters													
Week of gestation	21	39.182	39.000	37.000	41.000	1.168	19	38.889	40.000	37.000	41.000	1.900	0.677
Age (years)		30.211	31.000	18.000	43.000	6.294		31.571	32.000	22.000	38.000	3.936	0.482
Height (cm)		167.105	168.000	158.000	175.000	4.875		166.500	165.000	159.000	180.000	5.854	0.748
Body weight before pregnancy		62.158	60.000	48.000	80.000	9.008		66.846	63.000	54.000	98.500	12.742	0.231
BMI before pregnancy		22.258	21.875	18.750	29.400	3.093		23.859	22.321	19.133	30.900	3.922	0.207
Current BMI		28.675	28.031	26.534	32.031	2.0278		26.887	28.630	21.551	30.483	4.2935	0.495
Current body weight (kg)		75.980	77.000	62.000	84.000	7.6829		73.967	76.500	61.000	82.000	8.886	0.786
CRP (mother) (mg/l)*	17	2.414	1.800	0.900	5.300	1.631	15	4.500	5.200	0.600	7.000	2.765	0.144
Glucose (mother) (mg/dl)*		85.667	90.000	74.000	93.000	10.214		80.000	77.500	57.000	108.000	24.536	0.727
WBC (mother) (× 109/l.)		10.686	10.260	6.870	15.270	2.441		10.904	10.265	8.080	18.400	2.959	0.867
HGB (mother) (g/dl)		12.194	12.250	10.800	13.400	0.686		12.022	12.150	10.010	14.300	1.219	0.616
PLT (mother) (× 109/l.)		233.889	227.500	150.000	377.000	65.757		232.857	255.500	153.000	287.000	50.863	0.962
Newborn parameters													
CRP (child) (mg/l)	17	5.835	0.900	0.600	41.100	11.737	15	6.842	2.950	0.600	33.200	9.618	0.809
Glucose (child) (mg/dl)		64.622	64.750	38.000	88.800	14.054		59.700	61.850	29.600	80.700	13.606	0.349
Total bilirubin (child) (mg/dl)		7.101	7.610	0.090	11.200	3.368		9.027	8.695	0.630	19.070	4.684	0.208
WBC (child) (× 109/l.)		16.274	14.415	7.540	28.710	6.858		15.738	13.930	6.900	27.460	5.987	0.823
HGB (child) (g/dl)		17.572	18.150	13.200	20.700	1.990		17.008	16.600	14.300	20.400	1.856	0.430
PLT (child) (× 109/l.)		283.000	289.000	181.000	362.000	56.741		293.769	306.000	178.000	394.000	68.825	0.637
Body weight (child) (g)		3520.000	3600.000	2680.000	4280.000	422.058		3521.667	3585.000	2560.000	4190.000	433.754	0.992
Body length (child) (cm)		55.000	54.000	51.000	60.000	3.536		54.000	54.500	50.000	56.000	2.449	0.593
Cord blood parameters													
pH	8	7.300	7.330	7.190	7.400	0.091	8	7.313	7.340	7.150	7.360	0.074	0.793
BE		− 3.920	− 3.600	− 7.500	− 0.700	2.665		− 4.614	− 5.000	− 10.200	0.100	4.007	0.744
LAC arterial (mmol/l)		4.760	4.900	1.500	7.200	2.343		3.100	1.800	1.600	8.100	2.501	0.272
LAC venous (mmol/l)		0.833	1.000	0.300	1.200	0.4726		1.433	1.100	0.70	2.500	0.9452	0.381

N* Some parameters were not determined in the entire study group.

**Table 2:** Descriptive statistics regarding the study and control groups, regarding the type of delivery, order of pregnancy, gender of the newborn, vaccination against COVID-19 and the occurrence of gestational diabetes and hypothyroidism.

Parameter	Group of patients		Chi-square	*p*-value
	Control group	Patients with COVID-19 during pregnancy		
	N = 21	N = 19		
Delivery				
Cesarean section	11 (52%)	12 (63%)	0.474	0.538
Vaginal birth	10 (48%)	7 (37%)		
Number of pregnancies				
1	5 (24%)	7 (37%)		
2	9 (43%)	8 (42%)	1.113	0.573
3+	7 (33%)	4 (21%)		
Hypothyroidism in pregnancy				
Yes	2 (10%)	4 (21%)		
No	19 (90%)	15 (79%)	1.040	0.398
Gestational diabetes				
Yes	2 (10%)	4 (21%)	1.040	0.398
No	19 (90%)	15 (79%)		
Vaccination against COVID-19 during pregnancy				
Yes	2 (10%)	3 (16%)	0.385	0.654
No	19 (90%)	16 (84%)		
Gender of newborn				
M	11 (52%)	10 (53%)		
F	10 (48%)	9 (47%)	0.000	1.000

The study was conducted between February 2022 and October 2022, a period characterized by the dominance of the Omicron variant. Maternal COVID-19 severity was classified according to the NIH COVID-19 Treatment Guidelines. All patients in the COVID-19 group (N = 19) presented with mild illness. None of the participants required hospitalization or oxygen therapy. Regarding immunization during pregnancy, 16% of the COVID-19 group and 10% of the control group were vaccinated (Table 2). Data regarding pre-pregnancy vaccination status were incomplete for the cohort, reflecting regional trends during the study period.

### Assessment of the expression of the studied miR-155-5p, miR-21-3p and *SOX1* gene

The expression of the *SOX1* gene and miR-155-5p and miR-21-3p was assessed by qPCR in 40 samples of a heterogeneous population of human colostrum cells. PCR results were obtained in all samples. Based on the analyses, it was shown that the expression of the *SOX1* gene*,* miR-155-5p and miR-21-3p are statistically significantly higher in colostrum samples obtained from patients who experienced symptomatic COVID-19 infection during pregnancy. In the case of the *SOX1* gene, expression higher more than threefold (*p* = 0.006), miR-155-5p higher more than twice (*p* = 0.014), and miR-21-3p higher more than tenfold (*p* = 0.034) in the group of patients after COVID-19 compared to patients from the control group (Table 3).

**Table 3:** Relative expression (RQ) of the *SOX1* gene and miR-155-5p and miR-21-3p in colostrum in subgroups depending on the occurrence of COVID-19 infection during pregnancy.

*SOX1* gene and miRNA expression							
Parameter	Control group			Patients with COVID-19 during pregnancy			*p*-value
	N	Mean	SE	N	Mean	SE	
miR-155-5p	21	0.355	0.158	19	0.886	0.246	0.014*
miR-21-3p		0.247	0.103		2.764	1.390	0.034*
*SOX1*		12.946	2.315		39.813	13.103	0.006*

*p < 0.05 Student’s T-test. Statistically significant p-values (*p* < 0.05) are indicated in bold.

The relationship between *SOX1* gene expression and the tested miRNAs in colostrum with clinical parameters and laboratory test results for the patient and child was assessed. The analysis confirmed the existence of the following relationships:

Considering the entire study group (N = 40), significant positive correlations were noted between *SOX1* expression and miR-21-3p (*r* = 0.392 *p* < 0.05) and miR-155-5p and miR-21-3p (*r* = 0.882 *p* < 0.05), it was also observed that the expression of the *SOX1* gene statistically significantly negatively correlates with base deficiency (BE) in umbilical cord blood (*r* = -0.471 *p* < 0.05) and positively with the CRP level in the child (*r* = 0.331 *p* < 0.05) and lactate concentration in arterial cord blood (LAC arterial) (*r* = 0.442 *p* < 0.05) (Table 4).

**Table 4:** The occurrence of correlations between gene and microRNA testing and selected parameters characterizing the study group and the control group.

Parameter	Whole group N = 40			Patients with COVID-19 during pregnancy N = 19			Control group N = 21		
	RQ miR-155-5p	RQ *SOX1*	RQ miR-21-3p	RQ miR-155-5p	RQ *SOX1*	RQ miR-21-3p	RQ miR-155-5p	RQ *SOX1*	RQ miR-21-3p
RQ miR-155-5p	1.000	0.325	0.882*	1.000	0.400	0.400	1.000	− 0.244	0.514
RQ *SOX1*	0.325	1.000	0.392*	0.400	1.000	0.600	− 0.244	1.000	− 0.349
RQ miR-21-3p	0.882*	0.392*	1.000	0.400	0.600	1.000	0.514	− 0.349	1.000
Number of pregnancies	− 0.177	0.018	− 0.256	− 0.144	0.464	− 0.397	0.244	− 0.456*	0.338
Week of gestation	0.043	0.175	0.048	0.260	0.365	− 0.184	− 0.354	0.486	− 0.331
Age (years)	− 0.183	0.161	− 0.090	− 0.375	0.307	0.051	− 0.340	− 0.200	0.765*
Height (cm)	0.041	− 0.007	0.048	0.300	− 0.070	0.341	− 0.120	0.256	0.138
Body weight before pregnancy	− 0.126	− 0.051	− 0.124	0.104	− 0.004	− 0.211	− 0.786*	− 0.177	-0.057
BMI before pregnancy	− 0.165	− 0.040	− 0.158	− 0.036	0.093	− 0.380	− 0.857*	− 0.272	− 0.116
Current body weight (kg)	− 0.135	− 0.001	− 0.209	− 0.016	0.269	0.314	− 0.829*	0.045	− 0.504
HGB (mother) (g/dl)	− 0.045	− 0.204	− 0.135	0.127	− 0.383	− 0.182	− 0.342	− 0.251	− 0.497
WBC (mother) (× 109/l.)	0.199	0.200	0.089	− 0.294	0.340	− 0.225	0.335	0.096	− 0.230
PLT (mother) (× 109/l.)	0.080	0.095	0.174	− 0.070	− 0.068	0.333	0.627*	0.032	0.113
CRP (child) (mg/l)	− 0.108	0.331*	0.007	− 0.302	0.694*	0.165	− 0.359	− 0.158	− 0.379
Glucose (child) (mg/dl)	− 0.029	− 0.201	− 0.011	0.010	− 0.595*	0.646	0.272	0.016	0.162
Total bilirubin (child) (mg/dl)	0.149	− 0.114	0.225	0.275	− 0.191	0.289	0.278	− 0.702*	0.563
WBC (child) (× 109/l.)	− 0.131	0.044	− 0.138	0.030	0.119	− 0.477	− 0.416	− 0.214	− 0.330
HGB (child) (g/dl)	0.241	0.158	0.150	− 0.148	0.475	− 0.323	0.139	− 0.022	0.235
PLT (child) (× 109/l.)	0.191	− 0.129	0.255	− 0.101	− 0.459	0.386	0.318	− 0.210	0.007
PH	0.378	− 0.356	0.097	0.075	− 0.705	0.114	0.649	− 0.585	0.627
BE	− 0.105	− 0.471*	0.371	− 0.297	− 0.514	0.509	0.809	− 0.614	0.792
LAC arterial (mmol/l)	− 0.176	0.442*	− 0.264	− 0.141	0.778*	− 0.356	− 0.770	0.373	− 0.752
Body weight (child) (g)	0.124	0.066	0.201	− 0.197	0.151	0.420	− 0.623*	0.058	0.282

Pearson’s correlations *p < 0.05. Statistically significant Pearson’s correlation coefficients are indicated in bold. Positive values indicate a positive correlation, while negative values (preceded by a minus sign) indicate a negative correlation.

In the group of patients after symptomatic COVID-19 infection (N = 19), there was a significant relationship between *SOX1* gene expression and the CRP level in the child (*r* = 0.694 *p* < 0.05) and a negative relationship with the child’s glucose level (*r* = -0.595 *p* < 0.05) and a strong positive relationship with lactate concentration in arterial cord blood (LAC arterial) (*r* = 0.778 *p* < 0.05) (Table 4).

In the control group (N = 21), significant correlations were observed between miR-155-5p expression and body weight before pregnancy (*r* = -0.786 *p* < 0.05), BMI before pregnancy (*r* = -0.857 *p* < 0.05), current body weight (*r* = -0.829 *p* < 0.05), maternal PLT level (*r* = 0.627 *p* < 0.05) and newborn weight (*r* = -0.623 *p* < 0.05). In this group, *SOX1* expression significantly negatively correlated with the Number of pregnancies (*r* = -0.456 *p* < 0.05) and the child’s total bilirubin level (*r* = -0.702 *p* < 0.05). In turn, miR-21-3p positively and significantly correlated with maternal age (*r* = 0.765 *p* < 0.05) (Table 4).

## Discussion

Human milk is a complex biological fluid that serves as a dynamic conduit for immunological programming during early infancy, with its microRNA profile reflecting maternal immune adaptations to viral infections. Our findings reveal significantly elevated expression levels of miR-21, miR-155, and SOX1 in colostrum (collected on postpartum day 3) from mothers with SARS-CoV-2 infection during pregnancy compared to uninfected controls. Notably, a strong positive correlation between miR-155 and miR-21 expression suggests coordinated secretion of these regulatory microRNAs into human milk, potentially reflecting an amplified antiviral immune response in early lactation.

To our knowledge, this is the first study to evaluate SOX1 expression in colostrum in the context of maternal COVID-19 incidence during pregnancy.

Regarding the relationship between biochemical parameters and disease severity, our study focused on a clinically homogeneous cohort of non-hospitalized patients. Since all participants experienced a mild course of infection, no further stratification by severity was applicable. However, the fact that significant alterations in *SOX1,* miR-155, and miR-21 expression were observed even in these non-severe cases suggests that significant molecular alterations in colostrum occur regardless of the clinical intensity of the respiratory symptoms.

Our correlation analysis indicated that *SOX1* expression in the milk of mothers with a history of SARS-CoV-2 infection was significantly associated with several neonatal clinical markers, showing positive associations with CRP and arterial umbilical cord lactate (LAC), and a negative association with glucose levels. While these preliminary findings require validation in larger cohorts, they suggest a potential link between colostrum *SOX1* expression and the intensity of the neonatal systemic inflammatory response or metabolic stress. In the scientific literature, increased *SOX1* levels are often interpreted as part of the host defense mechanism in viral diseases, particularly those inducing strong inflammation or neurological involvement^46–48^. For instance, SARS-CoV-2 infection has been shown to alter the expression of genes within the hematopoietic system, including *SOX1*^26^, and clinical evidence suggests a link between COVID-19 and anti-SOX1 autoimmunity^49^.

The specific associations observed in our study further suggest that *SOX1* may serve as a molecular sensor of the perinatal biochemical environment. The positive correlation with LAC aligns with findings by Shen et al., who demonstrated that *SOX1* levels increase under hypoxic conditions^50^. Simultaneously, the negative correlation with glucose is consistent with reports that high glucose concentrations can reduce cellular differentiation toward neurons^51^. Although causal relationships cannot be directly inferred from this observational study, elevated *SOX1* expression in milk–combined with neonatal hypoglycemia and acidosis–might represent a maternal compensatory pathway aimed at supporting infant neural maturation and protecting progenitor cell populations in response to infection-induced stress^37–39^. This hypothesis is further supported by the observation that even the Omicron variant, despite its milder clinical course, induces a persistent molecular reconfiguration of the colostrum profile similar to SOX-family shifts observed in tissue repair processes^52^.

Additionally, we demonstrated elevated levels of miR-155-5p and miR-21-3p in the milk of women who experienced SARS-CoV-2 infection during pregnancy. This is consistent with other reports^53–56^. It is observed that disturbances in the regulation of miRNA levels occur in COVID-19 and are likely related to factors occurring during the infection^53–56^. SARS-CoV-2 infection has been shown to induce a robust inflammatory response, characterized by high cytokine levels and increased miR-155 and miR-21^57^. These miRNAs, acting as inflammatory mediators, may simultaneously suppress neurogenesis or alter progenitor cell phenotype, contributing to the neurological complications of COVID-19^53,55,56,58^.

In the scientific literature, miR-155 has been reported to be induced following TLR activation^59,60^, and its aberrant expression has also been observed in COVID-19 cases^53^. miR-155 is a well-established pro-inflammatory microRNA induced through TLR/NF-κB signaling pathways and has been widely implicated in antiviral immune responses^61–63^. miR-21 is commonly described as an anti-inflammatory regulator that modulates cytokine production and immune homeostasis^53,64–66^.

Keikha et al., also found that the relative expression of pro-neuroinflammatory miRNAs like miR-155 was increased in COVID-19 patients as the disease stage increased^53^. Hauroun et al. note that miR-155 plays a key role in the pathogenesis and severity of COVID-19 and may also be a good clinical diagnostic biomarker in disease detection and assessment of infection severity^55^. Bautista-Becerril et al. showed that increased levels of miR-21-5p expression are associated with worse COVID-19 outcomes in younger hospitalized patients^56^. The authors indicate that the overexpression of miR-21 and miR-155 results from targeting genes associated with the host response to SARS-CoV-2^53^. Interestingly, a study by Redenšek Trampuž et al. identified overlapping miRNAs in COVID-19 and neurodegenerative diseases, which may have valuable potential in predicting neurodegeneration in COVID-19 patients^58^.

Both miR-155 and miR-21 are indicated as microRNAs that play important roles in breast milk, primarily in the modulation of the immune system and metabolic control^67,68^. The role of miR-21 and miR-155 in breast milk is crucial for the proper development of the newborn’s immune and metabolic systems, and their stability is ensured by transport within exosomes.

Due to the physiological changes that occur during pregnancy, pregnant women are at increased risk of severe disease and poorer outcomes during SARS-CoV-2 infection^69–71^. Saulle et al. assessed the miRNA profile in the plasma and placenta of pregnant women infected with SARS-CoV-2. The authors showed that SARS-CoV-2 infection was associated with increased expression of certain miRNAs, including miR-155 and miR-21. Additionally, the authors note that miR-21 is highly expressed in human placentas of patients with preeclampsia; abnormal miR-21 expression has been identified as associated with fetal hypoxia and fetal growth restriction^72^. Graciliano et al.’s review details that maternal exposure to SARS-CoV-2 promotes changes in the composition of breast milk, causing distinct patterns of chemokines, cytokines, and growth factors^73^. SARS-CoV-2 infection during pregnancy impacts not only the mother’s overall health but also the functioning of her mammary glands. Chutipongtanate et al.’s study demonstrated that such infection alters the structure and content of extracellular vesicles in milk, which may disrupt or modify the natural exchange of biochemical information between a breastfeeding woman and her offspring^74^.

In the control group, miR-155-5p was negatively correlated with maternal pre-pregnancy weight, BMI, and neonatal birth weight. This aligns with Mazloom et al., who reported similar negative correlations in peripheral blood^75^. Mechanistically, miR-155 regulates adipocyte differentiation, its overexpression reduces brown adipose tissue mass, which may explain the link with neonatal weight^76,77^. While reduced miR-155 is associated with metabolic syndrome^78^, some reports show a positive correlation with adult obesity and macrophage expression^78,79^, or indicate that miR-155 deletion prevents obesity in mice^80^. Notably, these metabolic correlations were absent in our COVID-19 group, suggesting that infection may disrupt the typical relationship between miR-155 and body weight.

### Future directions

Our preliminary observations of higher *SOX1*, miR-155 and miR-21 expression might suggest a potential compensatory response in the maternal–fetal axis following COVID-19 infection. Although the exact biological impact remains to be elucidated, these changes affect molecules with established roles in immunity and neural plasticity. Breast milk has been described as a dynamic system of epigenetic communication; our results provide associative evidence that this system may undergo molecular reconfiguration in convalescent mothers. While it is tempting to speculate that increased marker expression could represent an adaptive response potentially influencing infant epigenetic regulation, such a hypothesis requires further validation. The observed interaction between the inflammatory environment and gene expression in milk raises exploratory questions about the long-term impact on neonatal development, which should be addressed in prospective studies including clinical infant outcomes.

Whether increased *SOX1* expression reflects a compensatory function protecting the nervous system against infection-induced inflammation remains a key exploratory question for future research.

### Strengths and limitations

Our study sheds new light on the molecular consequences of prenatal exposure to the SARS-CoV-2 virus, as manifested in the composition of human milk. The significant upregulation of microRNAs (miR-21, miR-155) and the transcription factor *SOX1* observed suggests that maternal infection during pregnancy triggers a signaling cascade that extends beyond the timeframe of the disease itself and modifies the biochemical profile of milk during different stages of lactation. The presented analyses are also the first reports of this type on the impact of COVID-19 during pregnancy on *SOX1* expression levels in human milk, with clinical correlations demonstrated. It should be noted that by mid-2022, when this study was conducted, the study population potentially possessed varying degrees of hybrid immunity from prior infections or pre-pregnancy vaccinations. This profile is representative of the real-world clinical landscape of the later pandemic phases, although specific history of infections occurring before the current pregnancy was not formally documented in this cohort.

However, it should be noted that our study has certain limitations. First, the analysis was conducted on a relatively small study group, suggesting the need to validate the obtained results in a larger cohort of patients with confirmed SARS-CoV-2 infection to increase the statistical power of our conclusions. Second, the assessment of *SOX1* gene expression was limited to transcript level analysis. Due to the lack of assays at the protein level, future studies should include immunoenzymatic or Western Blot analyses to confirm the translation and functional activity of the SOX1 protein in the tested samples. A limitation of this study is also the lack of specific data regarding the trimester of SARS-CoV-2 infection, which could influence the magnitude of the observed molecular changes. Furthermore, the use of a single validated reference gene (*GAPDH* for mRNA and RNU48 for miRNA) normalization is a limitation, however, its stability was confirmed across all experimental groups in our pilot cohort. The control group was selected based on negative history and clinical criteria, however, asymptomatic infections were not ruled out by serological testing, which is a potential confounding factor. Due to the relatively low vaccination rates in both groups, the influence of vaccination status could not be fully assessed. Furthermore, this study lacks longitudinal clinical data on infant development. Therefore, any implications regarding the neurodevelopmental protection or epigenetic modulation of the newborn remain speculative and require long-term follow-up studies. As an observational study, our results demonstrate associations rather than causal relationships between maternal COVID-19 history and colostrum molecular profiles.

## Conclusion

This study indicates that maternal SARS-CoV-2 infection during pregnancy is associated with a persistent alteration of the molecular profile of early postpartum milk. The observed upregulation of miR-21, miR-155, and *SOX1* suggests that the mammary gland undergoes long-term reconfiguration in response to prenatal inflammatory stress. Our findings highlight the role of human milk as a dynamic system of epigenetic communication that may relay infection-related signals to the infant. While the increased expression of these immunomodulatory and neurogenic markers potentially reflects a maternal compensatory mechanism, further longitudinal studies are required to determine their functional impact on neonatal clinical outcomes and long-term development.

## Supplementary Information

Below is the link to the electronic supplementary material.

Supplementary Material 1

## Data Availability

The datasets generated and/or analysed during the current study are available in the Figshare repository, 10.6084/m9.figshare.32038404
