# Supplementary material for: Higher expression of SOX1, miR-155, and miR-21 in the colostrum of SARS-CoV-2-infected mothers
Source: Sci Rep. 2026 Apr 20;16:18155. doi: 10.1038/s41598-026-49964-4 (PMC13253869; doi:10.1038/s41598-026-49964-4)
Supplement: Supplementary file 1 — Supplementary Material 1 [file 41598_2026_49964_MOESM1_ESM.docx]

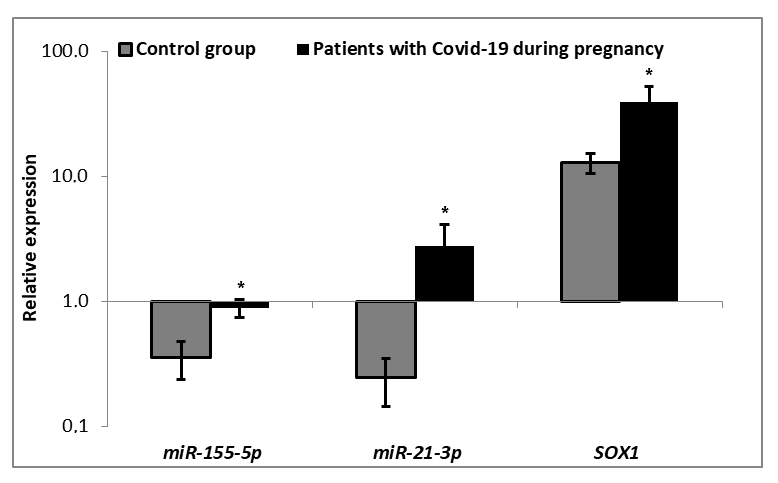


**Supplementary Figure 1.** Mean relative expression of the *SOX1* gene and miR-155-5p and miR-21-3p in colostrum in subgroups depending on the occurrence of COVID-19 infection during pregnancy logRQ±SE (graph presented on a logarithmic scale). *p<0.05 Student's T-test
